# SCMamba-SAM: transferring knowledge from natural images to medical tasks

**DOI:** 10.3389/fneur.2026.1938737

**Published:** 2026-10-15

**Authors:** Ye Tian, Yufei Yuan, Lu Wang, Xinming Yang, Yifeng Wu, Xu Xu

**Affiliations:** 1 Medical School, Tianjin University, Nankai, Tianjin, China; 2 The First Affiliated Hospital of Hebei North University, Zhangjiakou, China; 3 Graduate School of Tianjin Medical University, Tianjin, China; 4 College of Computer Science and Artificial Intelligence, Fudan University, Shanghai, China; 5 Tencent Jarvis Lab, Shenzhen, China; 6 Jilin Normal University, Siping, China

**Keywords:** deep learning, low-rank adaption, mamba, medical image segmentation, segment anything model

## Abstract

**Background and objective:**

The Segment Anything Model (SAM) has drawn significant attention because of its powerful performance in generating high-quality objectmasks. Despite its superior performance in handling natural images, SAM exhibits significant performance degradation when applied to medical images due to the substantial domain gap between natural and medical images. In addition, the specific characteristics of medical images, such as strong heterogeneity, low contrast, and wide individual variation, further raise dificulties in segmenting medical images.

**Methods:**

To bridge the semantic gap between natural and medical images, we propose an adaption framework, named SCMamba-SAM. It employs a foundational image encoder constructed by integrating the pre-trained encoder of SAM and Low-Rank Adaption technique. The Encoding Mamba Adapter adopts the spatial- and channel-Mamba blocks to capture task-related global features. Additionally, an enhanced mask decoder with deep supervision has been developed to eliminate the requirement for a prompt encoder, thereby transforming SAM into a fully automated and highly accurate image segmentation model.

**Results:**

Extensive experiments were performed on six public lesion segmentation datasets in different imaging modalities and body parts. The proposed SCMamba-SAM not only achieved performance superior to that of SOTA methods, quantitatively and qualitatively, but also exhibited high generalization.

**Conclusion:**

SCMamba-SAM holds promise as a useful tool for clinicians to diagnose and quantify lesions, providing important information for treatment decisions and surgery planning.

## 1 Introduction

Image segmentation is a fundamental task in computer-aided diagnosis, aiming to identify regions of interest (ROIs) within medical images, such as human organs, anatomical structures, and pathological lesions. Accurate segmentation provides essential qualitative and quantitative information for disease diagnosis and treatment planning. However, it is highly time-consuming and dependent on the operator's experience, leading to significant variability. Therefore, an automatic segmentation method is urgently needed to achieve more accurate, robust, and efficient results. Despite this need, the inherent heterogeneity, low contrast, and individual variability in medical images make automatic segmentation extremely challenging in clinical applications.

To address these challenges, deep convolutional neural networks (CNNs) have been extensively explored for medical image segmentation, significantly promoting the technique's progress and segmentation performance (1). Among the various CNN-based segmentation frameworks, UNet and its variants are the most widely adopted (2). Indeed, nnUNet, which utilizes a self-adaptive encoder-decoder architecture, has achieved state-of-the-art (SOFT) performance in a range of segmentation tasks (3). Due to their local receptive fields, CNNs also showed inherent limitations when addressing complex targets, especially in the segmentation of lesions characterized by high heterogeneity and low contrast between lesions and surrounding tissues.

Recently, more attention has been paid to Transformer-based architectures, such as the Vision Transformer (ViT) (4). Unlike CNNs, ViT leverages self-attention mechanisms to capture global dependencies of tokens within images, showing superior capability in medical image segmentation. ViT-based models, such as nnFormer (5), UNETR (6), and SwinUNet (7), illustrate the significant enhancements in model capabilities through the integration of Transformer modules. However, training ViT-based methods requires a substantial amount of labeled data. Trained on the SA-1B dataset, which contains 11 billion high-quality and diverse object masks generated from 11 million high-resolution images, the Segment Anything Model (SAM) has been developed by Meta AI (8). It has revitalized the field of image segmentation. Specifically, SAM utilizes a transformer-based image encoder and prompt encoder to convert images and various prompts (e.g., boxes, points, text, and masks) into embeddings that extract global semantic features. Subsequently, these features are fused in a lightweight mask decoder to predict object masks. To date, SAM has demonstrated superior performance in zero-shot segmentation and applicability across various scenarios (9). Although experiments have shown that SAM performs well in segmenting organs with clear boundaries, it struggles with amorphous regions, especially lesions with low contrast. For example, the effectiveness of SAM in polyp segmentation has been evaluated (10), but its performance still lags behind current SOTA methods. Huang et al. (11) built a new dataset consisting of 1,050K 2D medical images and 6,033K masks to comprehensively evaluate the performance of SAM on medical tasks. Although it is remarkably effective for certain objects, the randomness of point and box prompts can lead to unstable and sensitive results. Although several large-scale medical-imaging datasets are available, obtaining medical data with the diversity, independent case volume, and expert pixel-level annotations comparable to natural-image foundation-model datasets remains costly and task dependent. An alternative approach is to fine-tune weights pre-trained on SAM, trying to bridge the semantic gap between natural and medical images. Therefore, a novel SAM-based adaption framework, named SCMamba-SAM, is proposed based on the following two main considerations.

First, the complexity of medical data, coupled with its scarcity, presents unique challenges when applying Transformers for training on medical images, often leading to issues of over-fitting. To mitigate this problem, we proposed the use of Mamba, which effectively captures long-range dependencies during the flattening process. We have redesigned Mamba as a novel adapter to facilitate feature extraction from small-sample medical datasets for Transformers.

Second, SAM was redesigned with a strong emphasis on the importance of prompts, offering four types: point, box, text, and mask. These prompts are essential for SAM to achieve precise image segmentation. However, utilizing prompts requires substantial computational resources and considerable human effort when a large volume of samples needs to be tested. To address this challenge, we implemented deep supervision (DS) to fuse feature maps at different scales, allowing the model to focus effectively on the regions requirin segmentation.

We previously proposed the Spatial–Channel Mamba-adapted Segment Anything Model (SCMamba-SAM) method to explore the capacity of SAM in medical image segmentation [21].

Our main contributions are as follows. (**i**) We proposed an effective foundational adaption framework to achieve precise and automatic medical lesion segmentation. (**ii**) We constructed a foundational image encoder based on Low-Rank Adaption (LoRA) (12) technique with fewer trainable parameters to bridge the domain gap between natural and medical images. (**iii**) We introduced a novel Mamba-based adapter to enhance the representative capacity of features by capturing long-range dependencies between tokens and channels of feature maps at different levels. (**iv**) The proposed method not only achieved performance superior to that of the SOTA methods, quantitatively and qualitatively, but also exhibited high generalization on six public datasets in different imaging modalities and body parts.

## 2 Related work

### 2.1 Mamba in medical image segmentation

Mamba has been testified to be an effective method to accurately segment medical images by integration of local and global context. Although Vision Transformers are capable of processing global context and long-range dependencies, they are prone to overfitting when handling limited medical datasets. Recent advances in Structured State Space Models (SSMs) provide new insights for long-range modeling. Unlike transformers, Mamba can maintain its ability to model long-range dependencies while allowing for approximately linear expansion in sequence length. Ma et al. utilized two residual modules in conjunction with Mamba to re-architecture the encoder of a U-Net model (13), where the decoder consisted of transposed convolutions and residual blocks. Their U-Mamba achieved the best segmentation performance across four datasets. Liu et al. redesigned a Mamba-based network using ImageNet for pre-training (14). Their experimental results demonstrated that their network outperformed CNN-based, ViT-based, and SOFT Mamba-based models. Most previous studies have focused solely on simply replacing the Transformer architecture with Mamba, which incurs high retraining costs for the model. Mamba 2 proposed that combining Transformer and Mamba results in superior performance compared to using them individually (15). We initially attempted to utilize Mamba as an adapter to assist Transformer in feature extraction (16). Lately, Liang et al. proposed the organic integration of Vision Mamba as an adapter with Transformer for enhanced performance (17). Given the high complexity and strong heterogeneity of medical images, we redesigned Mamba by replacing all linear layers in Mamba with MLPs. In addition, we embedded Mamba within our encoder to assist the Transformer in handling long-sequence tasks while reducing the risk of overfitting due to low data availability.

### 2.2 Fine-tuning SAM in the medical field

The significant achievements of SAM in natural image segmentation have introduced a new benchmark model for medical image segmentation. However, the vast differences between natural and medical images often lead to a substantial performance decline when SAM has been applied directly to medical images. It even exhibited imperfections or total failures when dealing with segmentation targets with more challenging situations, such as weak boundaries, low contrast, small size, and irregular shapes (9, 18). To improve the unsatisfactory performance of SAM in medical image segmentation, more and more attention has been paid to refining SAM, including fine-tuning different SAM modules and training architectures analogous to SAM from scratch. As is known to all, full fine-tuning needs to update all parameters of SAM, which is a time-consuming, computationally intensive, and challenging process. Due to the limited availability of medical images and constraints on training resources, SAM typically employs various parameter-efficient fine-tuning techniques rather than full fine-tuning. Ma et al. fine-tuned the SAM image decoder using over one million medical masks while freezing the image encoder and prompt encoder (19). Zhang et al. introduced LoRA fine-tuning in the self-attention layers of SAM's encoder while maintaining the fine-tuning of the decoder to accomplish abdominal segmentation tasks (20). Wu et al. designed a novel encoder adapter, and fine-tuned only the adapter during the image encoder's fine-tuning process while keeping the rest of the image encoder frozen, thereby integrating medical knowledge into the encoder adapter (21). This method was proven to be effective across 19 datasets. To bridge the large domain gap between natural and medical images, Cheng et al. retained the fine-tuned encoder adapter while employing more comprehensive prompts (boxes, points, and masks) to assist SAM in segmentation. Although all of these methods showed improved performance, it is still a persistent challenge in SAM's zero-shot transfer to medical image segmentation (22). Therefore, we utilized LoRA for efficient fine-tuning to cooperate with a newly designed image encoder adapter, further extracting features to help SAM address its deficiencies in medical image segmentation. Therefore, we previously proposed the SCMamba-SAM method. To verify the outstanding performance of SCMamba-SAM in the field of medical image segmentation, we expanded into the following areas:

## 3 Method

The overall architecture of the proposed SCMamba-SAM is presented in Figure 1, which consists of a foundational image encoder, Encoding Mamba Adapters (EMA), and an enhanced mask decoder. Given a set of training samples {_*X*_*i*_, *Y*_*i*_}*i* = 1, 2, ..., *S*_, our goal is to obtain the optimal SCMamba-SAM model *F*(.) by maximizing the similarity between the prediction *P*_*i*_ = *F*(*X*_*i*_) and ground truth (GT) annotation *Y*_*i*_ of input image *X*_*i*_.

**Figure 1:**
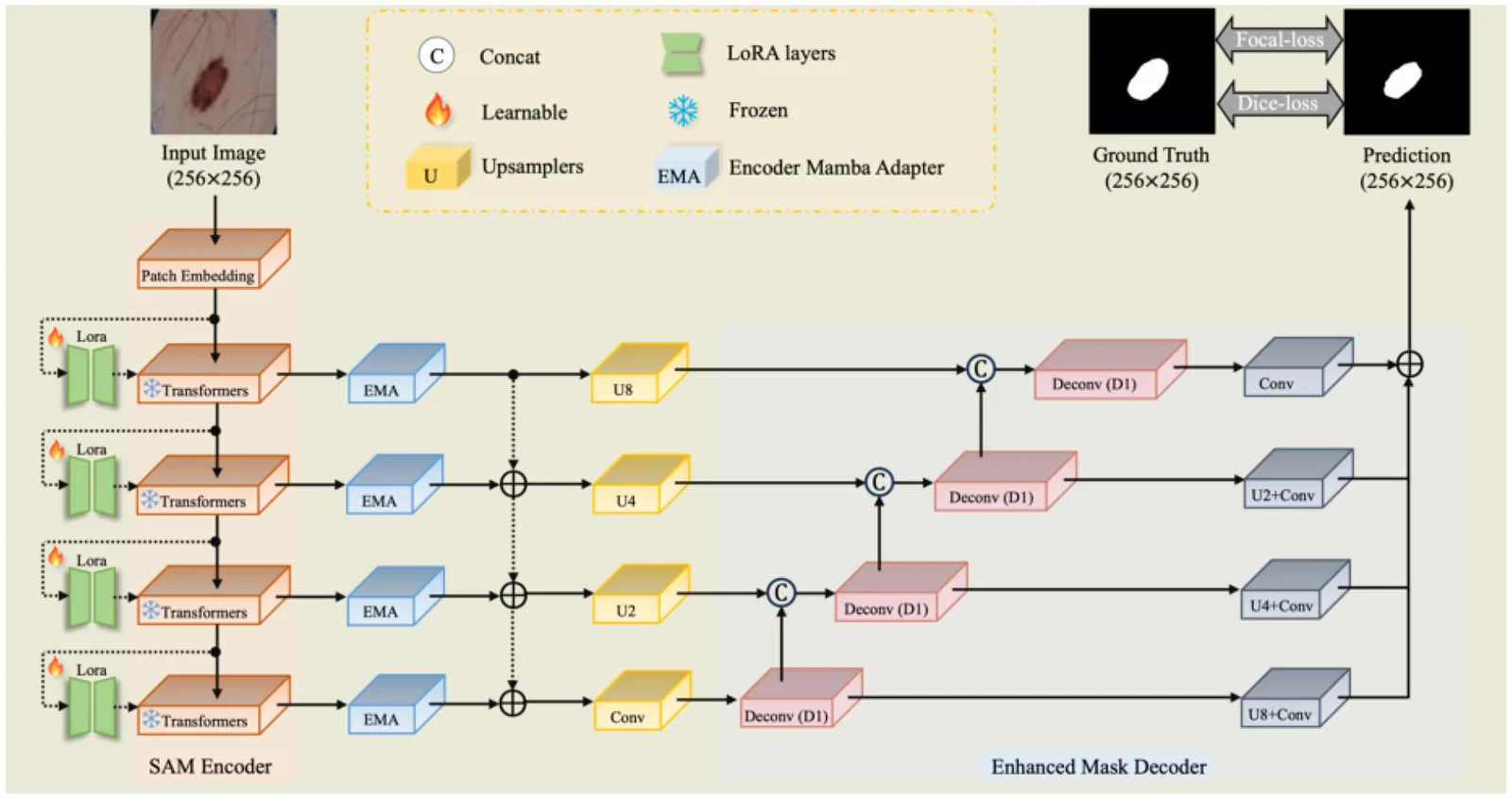
Architecture of the proposed SCMamba-SAM with encoder-decoder structure. While freezing the pre-trained encoder weights, Low-Rank Adaption (LoRA) layers are inserted to perform fine-tuning with fewer trainable parameters. Four Encoding Mamba Adapter (EMA) modules are incorporated to enhance feature maps at various depths within the image encoder. These enhanced feature maps are progressively fused in the enhanced mask decoder, which employs deep supervision to generate accurate target predictions.

The input image $X_{i}\in R^{C_{in}\times H\times W}$ is first fed into the foundational image encoder to extract abundant semantic features ${\left\{Z_{i}\in R^{N\times C}\right\}}_{i=1,2,...L}$. Here, *C*_*in*_ represents the channels of input image; *H* and *W* stand for image height and width; *L* is the number of layers of multi-head self-attention (MHSA) in foundational image encoder; $N=\frac{HW}{p^{2}}$ is token length after patch splitting with size *p* = 16, and *C* represents hidden size of each MHSA layer. Subsequently, feature maps *Z*_*i*_ at different depths are adapted by the EMAs to extract the fine-grained features related to the targets. These adapted features are fused step-wise in the enhanced mask decoder to generate predictions $P_{i}\in R^{C_{out}\times H\times W}$, where *C*_*out*_ is the number of target categories. Details of each component will be explained in the following sub-sections.

### 3.1 Foundational image encoder

The foundational image encoder retains the SAM's image encoder, which adopts the architecture of ViT-B, consisting of *L* = 12 MHSA layers. In order to tackle the significant semantic gap between natural and medical images, we leverage LoRA technology to indirectly fine-tune the image encoder efficiently with significantly fewer learnable parameters. Specifically, LoRA layers are inserted into each MHSA layer, as depicted in Figure 2. For ***Z***_*i*_ of *i*-th transformer block, the LoRA fine-tuning of the MHSA layer is formulated as follows:

$$\begin{array}{l} Q_{i}=W_{Q}\left(Z_{i}\right)+W_{Q}^{up}\left(W_{Q}^{down}\left(Z_{i}\right)\right), \end{array}$$ (1)

$$\begin{array}{l} K_{i}=W_{K}\left(Z_{i}\right), \end{array}$$ (2)

$$\begin{array}{l} V_{i}=W_{V}\left(Z_{i}\right)+W_{V}^{\text{up}}\left(W_{V}^{\text{down}}\left(Z_{i}\right)\right), \end{array}$$ (3)

$$\begin{array}{l} {\hat{Z}}_{i}=MHSA\left(Q_{i},K_{i},V_{i}\right)+Z_{i}, \end{array}$$ (4)

where ***W***_*Q*_, ***W***_*K*_, and ***W***_*V*_ are the frozen weights of the linear layers, for generation of the *Query*(*Q*), *Key*(*K*), and *Value*(*V*) matrices in each MHSA layer. Two trainable linear layers $W_{Q,V}^{\text{down}}\in R^{r\times C}$ and $W_{Q,V}^{up}\in R^{r\times C}$ inside of the LoRA layer reduce and restore the feature dimension, aiming to learn the updates of *Q*_*i*_ and *V*_*i*_ with less trainable parameters. Here, *r* represents a smaller projection dimension used in low-rank decomposition. The updated *Q*_*i*_ and *V*_*i*_, along with the original *K*_*i*_ are fed into the MHSA layer for self-attention computation. With the help of LoRA layers, we obtain adapted features ${\hat{Z}}_{i}$ of the medical image domain.

**Figure 2:**
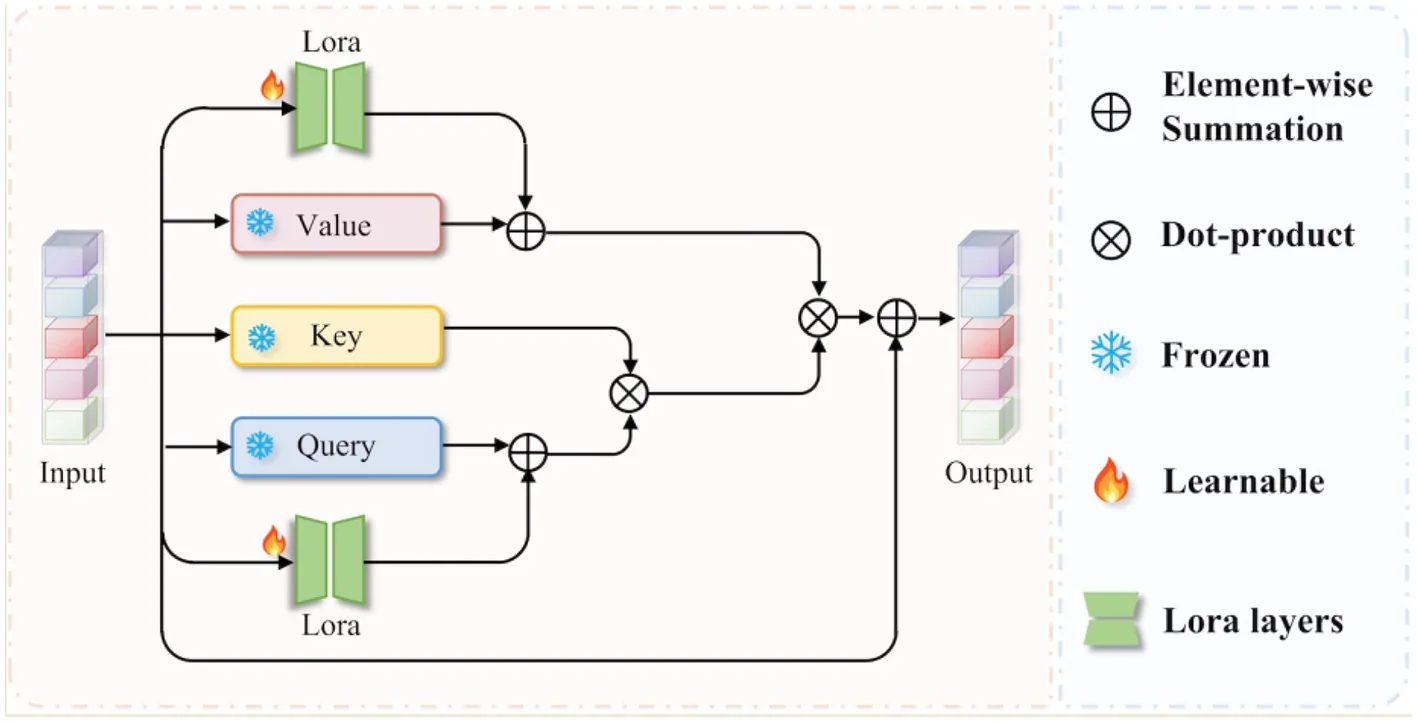
LoRA fine-tuning of a multi-head self-attention (MHSA) layer in a foundational image encoder. LoRA layers, each composed of two linear layers, are inserted into the MHSA layers with frozen weights to update the Query and Value matrices, achieving fine-tuning.

### 3.2 EMA architecture

Due to the large domain gap between natural and medical images, the direct application of pre-trained SAM in medical images usually performs poorly. Even fine-tuned with LoRA, it is still challenging to handle medical targets with high heterogeneity. To address this issue, we propose a novel EMA module to further enhance feature representations.

The architecture of EMA is demonstrated in Figure 3. EMA is composed of two sequential blocks, named Spatial-Mamba and channel-Mamba. Spatial-Mamba focuses on capturing the Token long-range inter-dependencies among tokens, whereas channel-Mamba is dedicated to building the global relationships among channels. Four EMA are plugged into the image encoder to enhance feature maps ${Ẑ}_{i}\in R^{N\times C}$ from *i*-th Transformer block, which are fused by addition:

$$\begin{array}{l} {Ê}_{i}=EMA\left({Ẑ}_{i}\right), \end{array}$$ (5)

$$\begin{array}{l} E_{i}=\underset{j\leq i}{\sum }{Ê}_{j},i,j=3,6,9,12. \end{array}$$ (6)

Inside of EMA, feature maps Ẑ_*i*_ are sequentially processed by Spatial-Mamba and channel-Mamba. Both blocks have the same architecture, consisting of a squeeze-MLP (multi-layer perception) layer, a Mamba layer, and an expand-MLP layer. The anterior squeeze-MLP and posterior expand-MLP in both blocks perform dimension reduction and restoration, respectively, aiming to decrease the computational load of the Mamba layer. Specifically, the token number is projected from *N* to *N*/8 in the Spatial-Mamba, and the token dimension is reduced from *C* to *C*/3 in the channel-Mamba. The transpose operation is inserted prior to and posterior to channel-Mamba, to exchange the token length and dimension.

**Figure 3:**
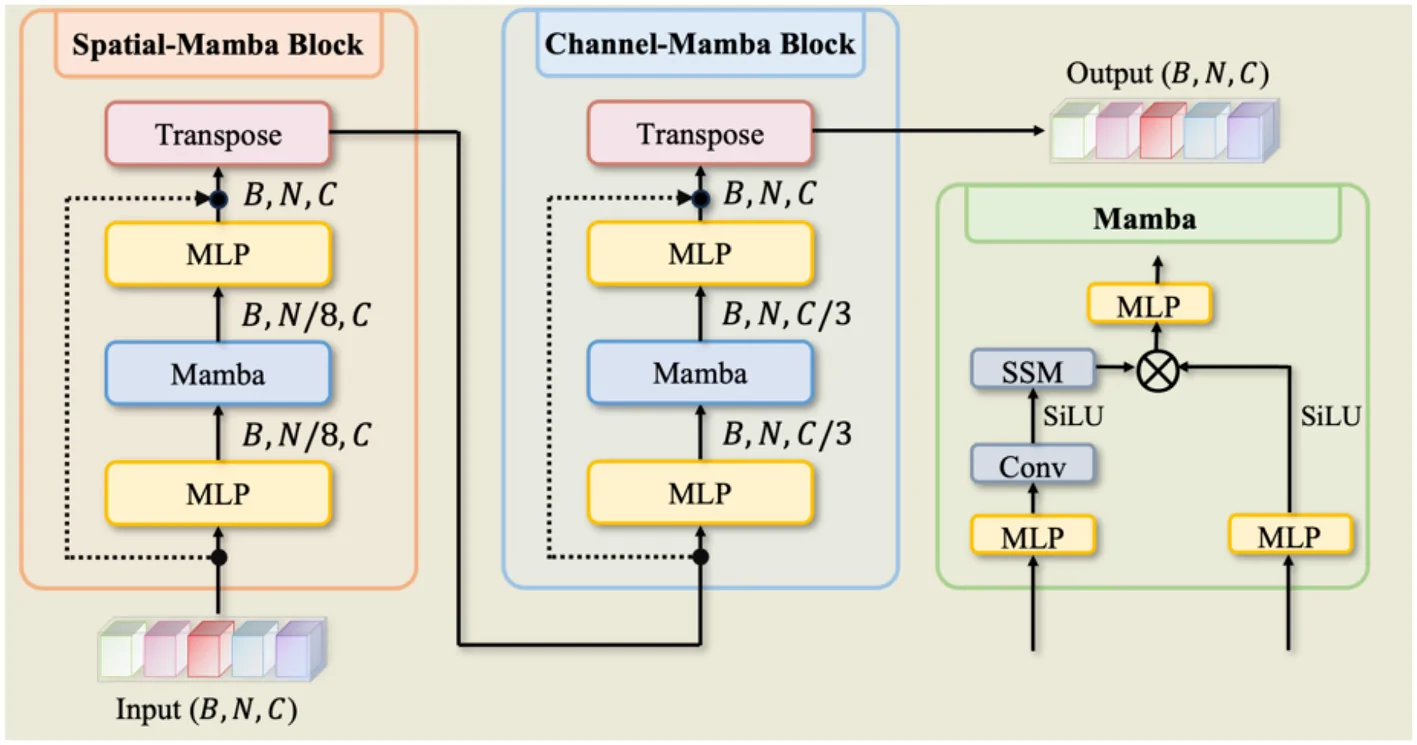
Architecture of the proposed EMA. The input tokens are sequentially processed by Spatial-Mamba and channel-Mamba to capture the global dependencies among tokens and channels.

Mamba layer is the core of EMA to build global dependencies of the token sequence (23). Based on the state space model (SSM) theory, Mamba is capable of capturing long-range dependencies of sequence data with linear complexity and has shown excellent performance in medical image-related tasks (24). Our Mamba layer consists of two parallel branches, as shown in the right part of Figure 3. In the first branch, input features are sequentially processed by an MLP layer, a convolutional layer, a SiLU activation layer, and the SSM layer. The second branch includes an MLP layer to expand input features, followed by a SiLU activation layer. The outputs of both branches are fused by Hadamard products, followed by another MLP layer to project the outputs back into the original shape. Different from the original Mamba layer, we replaced all the linear layers with MLPs to augment the capability of feature representations.

### 3.3 Enhanced mask decoder

Mask decoder is composed of four Deconv layers, each consisting of the up-sampling layer, ReLU layer, and Conv2D layer. Specifically, EMA features $E_{i=3,6,9,12}\in R^{N\times C},$ are first converted to $E_{i}\in R^{C\times \frac{H}{p}\times \frac{W}{p}}$, which are up-sampled and fed into Deconv layers for feature fusion and resolution restoration step-wise as illustrated in Figure 1. Here, *p* is the patch splitting size used in the foundational image encoder.

Deep supervision (DS) mechanism is used during training. The features of different Deconv layers *D*_*j* = 1, 2, 3, 4_ are fed into segmentation heads, each of them composed of an up-sampling layer to match the resolution of GT, and followed by a convolutional layer to convert feature channels to the number of target categories *C*_*out*_. A multi-scale weighted loss function *L*_*DS*_ is constructed by the weighted summation of the FocalDice-loss (25) between predictions and GT:

$$\begin{array}{l} L_{DS}=\underset{j}{\sum }w_{j}\cdot \left(L_{Dice}\left(P^{\left(j\right)},Y\right)+L_{Focal}\left(P^{\left(j\right)},Y\right)\right), \end{array}$$ (7)

where *L*_*Dice*_ and *L*_*Focal*_ represent the Dice loss and Focal loss, respectively; *w*_*j*_ are weights for *j*-th feature level; *P*^(*j*)^ is outputs of *j*-th segmentation head, whereas *Y* stands for GT. In practice, we set *w*_*j* = 1, 2, 3, 4_ = 1.

## 4 Experiments

### 4.1 Datasets

Six publicly available datasets across different imaging modalities and disease lesions are included as benchmark datasets. They are the ISIC2017 (26), ISIC2018 (27), Kvasir-SEG (28), TN-SCUI2020 (29), CT-ICH2020 (30), and MR-FCD2023 datasets (31).

### 4.2 Implementation details

The proposed SCMamba-SAM was implemented using Python 3.10 and PyTorch 2.1.0 on an Ubuntu 22.04 server equipped with an A800 80G GPU. In our SCMamba-SAM model, we utilized and froze the pre-trained weights of SAM in the foundational image encoder while randomly initializing the other model components. We chose Adam (32) optimizer with a learning rate of 0.0001 to train the proposed SCMamba-SAM. The total number of training epochs was 200 with a batch size of 8. The input images were uniformly resized to 256 × 256. Four evaluation metrics were chosen to assess the segmentation performances: the Dice coefficient (*Dice*) (33), Intersection over Union (*IoU*) (34), Precision, and Recall (35).

### 4.3 Comparison with other methods

We first conducted experiments to comprehensively compare our SCMamba-SAM with SOTA deep learning methods on ISIC2017. These SOTA methods are categorized into four groups: (1) CNN-based segmentation methods, including U-Net (36), U-Net V2 (37), R2U-Net (38), FAT-Net (39), BiONet (40), DeepLabv3+ (41), MB-DCNN (42), and SESV-Dlab (43); (2) Transformer-based methods, such as TransU-Net (44) and SwinU-Net (7); (3) Generative Adversarial Networks (GANs) based methods. Data Augmentation GAN [DAGAN (45)] is chosen for comparison due to its strong model performance in data-scarce scenarios; and (4) SAM-based methods, including the pre-trained models and fine-tuned models. For the pre-trained models, we chose the SAM (8), MedSAM (19), and SAM-Med2D (22), denoted as SAM*, MedSAM*, and SAM-Med2D*, respectively, due to their excellent generalization performance in dealing with natural and medical images. For fine-tuned models, SAM and another adaptation framework named MSA (21) were fine-tuned on the same training set of ISIC2017. Quantitative evaluation results of *IoU* and *Dice* on the testing set of ISIC2017 are provided in Table 1. In addition, we listed parameter count (*Params*) and the floating point operations (*GFLOPs*) for each model.

**Table 1:** Performance comparison of different methods on ISIC2017 dataset.

Method	IoU	Dice	Params	GFLOPs
U-Net (36)	0.699	0.796	30 M	42
U-Net v2 (37)	0.750	0.838	25 M	5
R2U-Net (38)	0.757	0.840	92 M	103
FAT-Net (39)	0.772	0.854	27 M	43
BiONet (40)	0.755	0.841	57 M	71
DAGAN (45)	0.771	0.859	56 M	62
DeepLabv3+ (41)	0.752	0.838	39 M	10
MB-DCNN (42)	0.760	0.843	53 M	55
SESV-Dlab (43)	0.753	0.839	46 M	30
TransU-Net (44)	0.765	0.850	100 M	25
SwinU-Net (7)	0.767	0.849	26 M	6
SAM^*^ (8)	0.550	0.676	90 M	744
SAM	0.712	0.795	90 M	64
MedSAM^*^ (19)	0.726	0.836	90 M	743
MSA (21)	0.737	0.829	94 M	70
SAM-Med2D^*^ (22)	0.736	0.842	271 M	130
**SCMamba-SAM (ours)**	**0.777**	**0.861**	**395 M**	**1,564**

^*^Indicates pre-trained model.

In order to further validate the performance of the proposed SCMamba-SAM, we conducted additional experiments on ISIC2018, Kvasir-SEG, TN-SCUI2020, CT-ICH2020, and the more challenging MR-FCD2023 dataset. Here, we focused on comparison among SAM-based methods, including the pre-trained models and fine-tuned models. For the pre-trained models, we chose the SAM*, MedSAM*, and SAM-Med2D*. For fine-tuned models, SAM and MSA were fine-tuned on the same training set of each dataset as that used by our method.

Figure 4 visualizes some representative samples and the lesion segmentation results by different methods for qualitative comparisons. We provide some representative samples of each dataset and corresponding segmentation results by different methods in Figure 5. The Figure 6 represents the quantitative comparisons of *DICE*, *IoU*, Precision, and Recall accomplished by different models on ISIC2018, Kvasir-SEG, TN-SCUI2020, CT-ICH2020, and MR-FCD2023 datasets.

**Figure 4:**
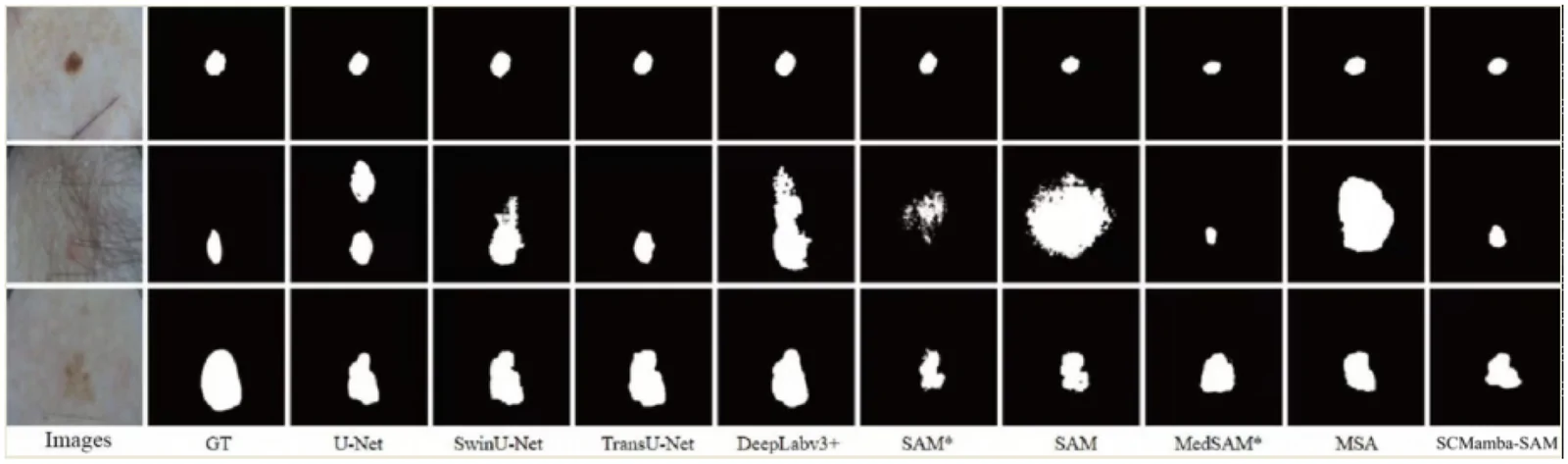
Visual segmentation comparison of SCMamba-SAM with SOTA methods on ISIC2017. *Denotes pre-trained model.

**Figure 5:**
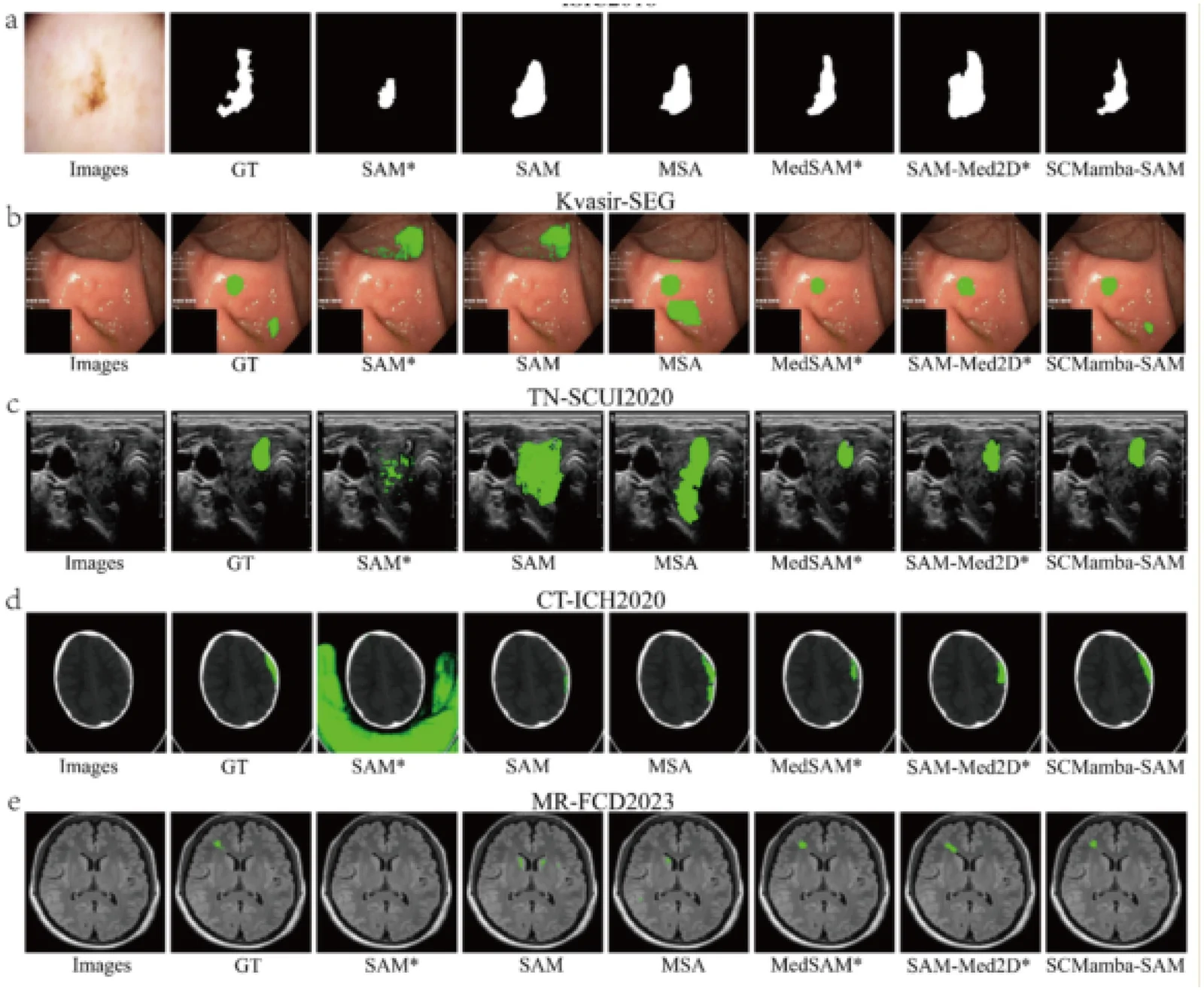
Visual segmentation comparison of SCMamba-SAM with SOTA methods on five public datasets. ^*^Denotes pre-trained model.

**Figure 6:**
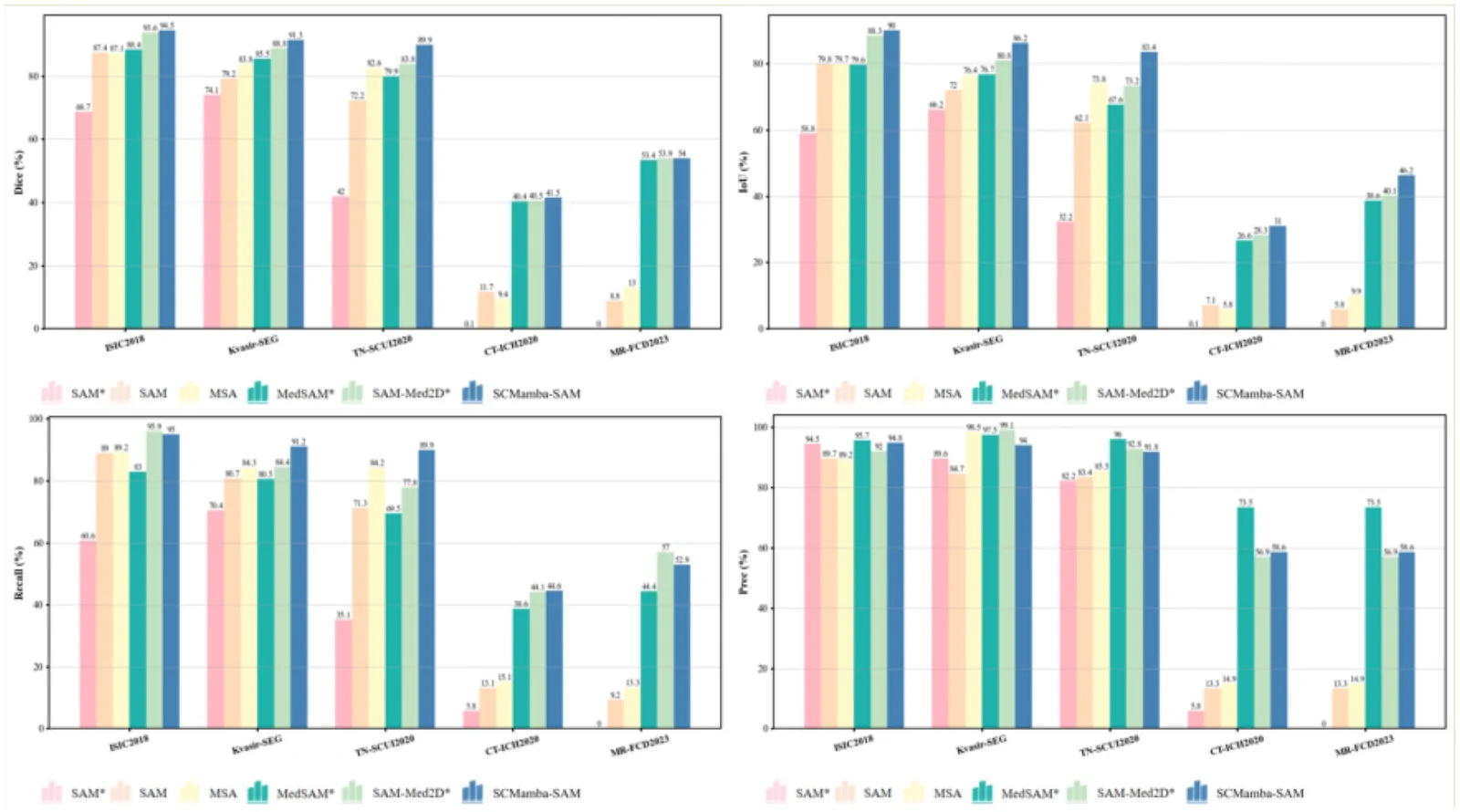
The quantitative metrics of Dice, IoU, Recall, and Prec across five public datasets. ^*^Denotes pre-trained model.

### 4.4 Ablation studies

We conducted extensive ablation experiments to validate the effects of key modules and hyperparameters of the SCMamba-SAM. For simplicity, all experiments were conducted on the ISIC2017 dataset. The details are presented below.

#### 4.4.1 Ablation experiments on different modules

The key modules of SCMamba-SAM are LoRA layers in the foundational image encoder, EMA for feature adaptation, and DS for multi-scale supervision in the decoder. The pre-trained SAM was set as the baseline. We sequentially added LoRA layers, DS, and EMA to obtain three different models. Each model was trained on the same dataset with the same parameters, and their performance was evaluated on the testing set using *IoU*, *Dice*, *Prec*, and *Recall* metrics, as listed in Table 2.

**Table 2:** Ablation experiment results of different modules in the proposed SCMamba-SAM on ISIC2017.

LoRA	DS	EMA	IoU	Dice	Prec	Recall
✗	✗	✗	0.550	0.676	0.863	0.645
✓	✗	✗	0.765	0.849	0.939	0.816
✓	✓	✗	0.765	0.850	0.932	0.824
✓	✓	✓	**0.777**	**0.861**	**0.946**	**0.825**

Baseline is the pre-trained SAM without Low-Rank Adaption (LoRA), deep supervision (DS), and Encoding Mamba Adapter (EMA). The ablation experiments demonstrated that our model achieved the best results.

#### 4.4.2 Ablation Study of EMA at different positions

EMA is embedded at four positions (3, 6, 9, 12-th transformer blocks) of the image encoder for multi-scale feature adaptation and fusion. We constructed four single-EMA versions of SCMamba-SAM by plugging *E*_3_, *E*_6_, *E*_9_, *E*_12_ into the baseline separately. Each model was trained on the same dataset with identical parameters, and their performance was assessed on the testing set using *IoU*, *Dice*, *Precision*, and *Recall*, as listed in Table 3.

**Table 3:** The ablation experiment results of EMA at different positions in the proposed SCMamba-SAM on ISIC2017.

E3	E6	E9	E12	Dice	IoU	Prec	Recall
✗	✗	✗	✗	0.848	0.754	0.943	0.820
✓	✗	✗	✗	0.831	0.738	0.953	0.818
✗	✓	✗	✗	0.856	0.770	0.958	**0.844**
✗	✗	✓	✗	0.857	0.775	0.956	0.834
✗	✗	✗	✓	0.858	0.776	**0.961**	0.839
✓	✓	✓	✓	**0.861**	**0.777**	0.946	0.825

The baseline does not use any EMA block. The ablation experiments demonstrated that our model achieved the best results.

#### 4.4.3 Ablation experiments on the structure of EMA

EMA consists of two serial components that extract features for Tokens and Channels. We conducted single-channel experiments for both aspects, resulting in two models for comparison with the serial EMA. Each model was trained on the same dataset with identical parameters, and their performance was evaluated on the testing set using *IoU*, *Dice*, *Prec*, and *Recall* for comparison with SCMamba-SAM, as listed in Table 4.

**Table 4:** The ablation experiment results of the proposed SCMamba-SAM on ISIC2017 regarding the EMA serial structure.

Channel	Token	Dice	IoU	Prec	Recall
✓	✗	0.856	0.774	0.951	**0.842**
✗	✓	0.858	0.773	**0.960**	0.834
✓	✓	**0.861**	**0.777**	0.946	0.825

The ablation experiments demonstrated that our model achieved the best results.

#### 4.4.4 Ablation experiments on Mamba

Mamba captures global feature dependencies in medical imaging. To enhance its performance, we replaced the linear layers in the original Mamba with MLPs and assessed the effectiveness. We compared MLP-Mamba with both Self-Attention and the original Mamba. The two serial Mamba layers in EMA were substituted with Self-Attention and the original Mamba, respectively, and the results were evaluated for comparison with SCMamba-SAM, as listed in Table 5.

**Table 5:** The ablation experiment results of the proposed SCMamba-SAM on ISIC2017 regarding the improvement of the Mamba layer in the EMA.

EMA	Dice	IoU	Prec	Recall
Self-Attention	0.815	0.713	0.876	0.817
Mamba-Linear	0.848	0.764	0.933	0.822
Mamba-MLPs	**0.861**	**0.777**	**0.946**	**0.825**

The ablation experiments demonstrated that our model achieved the best results.

### 4.5 Hyper-parameter analysis

#### 4.5.1 Effects of LoRA rank

LoRA is a low-rank fine-tuning method. It has been commonly reported that the rank *r* in LoRA layers could significantly affect the model's performance and scale. There is a trade-off between model performance and scale. Lower rank *r* would significantly decrease the model parameters and complexity at the cost of capability in feature presentation. In this study, we conducted an ablation experiment to evaluate the performance variations impacted by different *r* values. By varying *r*, we assessed *Params* and *GFLOPs*, as well as *Dice* and *IoU* on the testing set of ISIC2017. The results of the ablation experiments regarding the *r* values are presented in the Table 6.

**Table 6:** Comparative experimental results of different rank values in LoRA on ISIC2017.

Rank(*r*)	IoU	Dice	Params	GFLOPs
32	0.771	0.854	385M	1,550
64	0.766	0.851	388M	1,555
**128**	0.777	0.861	395M	1,564
256	0.774	0.857	407M	1,581
512	0.777	0.859	432M	1,616
1,024	0.780	0.862	482M	1,686

The values marked in bold are what we consider to be the ideal *r* values.

#### 4.5.2 Ablation on EMA configuration

There are two key hyperparameters in the configuration of EMA: the scaling factors of the token number and the token dimension. With the scaling factor of the token number, the squeeze-MLP in the Spatial-Mamba layer reduces the token number to decrease the computation burden. In a similar way, the token dimension is projected into a lower value in the channel-Mamba layer to decrease computation complexity. Both scaling factors would greatly affect the performance of the EMA. We conducted a series of experiments with different settings of scaling factors to validate their performances, further guiding us to choose the optimal configuration. The scaling factor of token number is set to [3, 6, 9], whereas the scaling factor of token length is set to [2, 4, 8]. There are 9 different configurations overall. We trained Mama-SAM with these EMA configurations, while others were kept the same. The evaluation results with *Dice* and *IoU* metrics are presented in Table 7.

**Table 7:** Ablation experiment results of different EMA configurations on ISIC2017.

Token dimension	Token number	IoU	Dice
/3	/2	0.767	0.853
/3	/4	0.776	0.860
**/3**	**/8**	**0.777**	**0.861**
/6	/2	0.773	0.857
/6	/4	0.766	0.852
/6	/8	0.766	0.850
/9	/2	0.765	0.849
/9	/4	0.766	0.850
/9	/8	0.762	0.849

The ablation experiments demonstrated that our model achieved the best results.

## 5 Discussion

Although SAM has achieved great success in natural image segmentation, issues arose when applying it to medical image segmentation due to the significant domain gap between natural and medical images. In order to bridge the domain gap and adapt SAM to medical images, a novel SCMamba-SAM framework is proposed in this article. The proposed SCMamba-SAM takes advantage of the powerful SAM's encoder to construct a foundational image encoder with embedded LoRA layers for domain adaptation with fewer learnable parameters. Moreover, EMA, leveraging the Mamba technique, receives different levels of features from a foundational image encoder to capture global dependencies and adapt to be task-specific, e.g., lesions with high heterogeneity and blurred edges. In addition, the usage of deep supervision in the decoder further enhances the performance in handling medical targets with varying shapes and sizes.

### 5.1 Performance of lesion segmentation

Extensive experiments have been conducted to validate the superior performance of our proposed SCMamba-SAM on six public lesion segmentation datasets. These datasets involve different imaging modalities and body parts.

#### 5.1.1 Skin lesion segmentation on dermatoscopy images

The segmentation performance was evaluated on ISIC2017 and ISIC2018. SCMamba-SAM achieved an *IoU* of 0.777 and a *Dice* score of 0.861 on ISIC2017, outperforming CNN-based, GAN-based, Transformer-based, and SAM-based methods. Qualitative comparisons in Figure 4 demonstrate that SCMamba-SAM's segmentation results closely match the original lesion contours, particularly highlighting the superiority in delineating finer details, unlike other methods. Similar results were obtained on ISIC2018, where SCMamba-SAM exhibited state-of-the-art performance with *IoU* = 0.900 and *Dice* = 0.945.

#### 5.1.2 Gastrointestinal polyp segmentation on endoscopy images

Evaluations on the Kvasir-SEG dataset revealed that SCMamba-SAM outperformed the second-ranking SAM-Med2D* by 6% in *IoU*, 3% in *Dice*, and 7% in *Recall*. As shown in Figure 5b, despite the varying sizes and numbers of polyps, SCMamba-SAM successfully segmented small polyps with greater accuracy than fine-tuned SAM and other models, which failed in this aspect.

#### 5.1.3 Thyroid nodule segmentation on ultrasound images

In experiments using the TN-SCUI2020 dataset, SCMamba-SAM demonstrated significant improvement over other SAM-based methods, achieving an *IoU* of 0.834 and outperforming the second-ranked MedSAM* by 10%. It also exceeded other methods by 5% to 6% in *Dice* and *Recall*. Visual comparisons in Figure 5c highlight SCMamba-SAM's ability to produce smooth boundaries in segmentations.

#### 5.1.4 Intracranial hemorrhage segmentation on CT images

SCMamba-SAM was validated using the CT-ICH2020 dataset, comprising images with five different hemorrhage types. It consistently surpassed other SAM-based methods, achieving a *Dice* score of 0.415, 1% higher than SAM-Med2D*. Its *IoU* performance was more pronounced, exceeding SAM-Med2D* by over 3%. Figure 5d illustrates two cases where SCMamba-SAM effectively segmented lesions, addressing the domain gap between natural and medical images.

#### 5.1.5 FCD lesion segmentation on MR images

Segmenting FCD lesions is challenging due to subtle structural changes in MR images, as depicted in Figure 5e. SCMamba-SAM outperformed other methods with an *IoU* of 0.462, 6% higher than SAM-Med2D*. Despite the complexity of FCD lesions, SCMamba-SAM successfully segmented MRI-negative cases, whereas other models struggled significantly.

### 5.2 Effectiveness of model architecture

Ablation experiment results listed in Table 2 illustrated the effectiveness of different modules in SCMamba-SAM. LoRA fine-tuning plays an important role in bridging the domain gap between natural and medical images. The *IoU* and *Dice* increase from 0.55 to 0.765, and 0.676 to 0.849, respectively. Next, with the help of DS, *Recall* increases from 0.816 to 0.824 while *IoU* and *Dice* remain the same. These results demonstrated that deep supervision could contribute to accurate recognition of more true positive lesion pixels. Lastly, when the proposed EMA is equipped, *IoU*, *Dice*, *Prec*, and *Recall* are all further improved, making SCMamba-SAM the SOTA method at the task of skin lesion segmentation on ISIC2017. It indicates that the proposed EMA would learn important features highly correlated to downstream tasks, thus augmenting the performance.

The results of the ablation experiments regarding the positions of EMA are presented in Table 3. We chose to remove both the EMA and DS components as our baseline model. When applying EMA to the output of the shallow layers of the transformer, the Dice coefficient decreased by 1.7%, and the Intersection over Union (IoU) decreased by 1.6% compared to the baseline model. In contrast, applying EMA to the output embeddings of the middle layers resulted in a significant increase of 1.6% in IoU and a 2.4% increase in Recall compared to the baseline model. For the embeddings from the ninth layer and the final layer, the improvements in Dice and IoU were only 0.1% compared to the middle layer embedding. When introducing DS, the combination of EMA from four layers achieved the best performance, resulting in a 1.3% increase in Dice and a 2.3% increase in IoU compared to the baseline model.

The results of the ablation experiment listed in Table 4 illustrated the effectiveness of the serial structure of the EMA. We conducted experiments by isolating the components dedicated to Token feature extraction and Channel feature extraction for comparison with our serial EMA structure. The results indicated that the performance degraded when only one of these components was used. Notably, with the Token-only EMA only, the Dice coefficient dropped by 0.5% and the IoU decreased by 0.3% compared to the serial EMA structure. Additionally, the Channel-Eonly MA exhibited significantly unstable performance in comparison to the serial structure.

The ablation results of the Mamba component in the EMA are presented in Table 5. We selected self-attention as the baseline model. Compared to the baseline model, the introduction of the initial Mamba resulted in a 3.3% increase in the Dice coefficient and a 5.1% increase in the Intersection over Union (IoU). Specifically, the incorporation of self-attention led to an information surplus, whereas the initial Mamba was effective in capturing long-range dependencies, thereby assisting the transformer in feature extraction. Moreover, the improved Mamba demonstrated an increase of 1.3% in both the Dice coefficient and IoU compared to the initial Mamba. This improvement is attributed to the replacement of the linear layers in Mamba with multi-layer perceptrons (MLPs), allowing the Mamba to extract more complex features.

### 5.3 Reasonable selection of hyperparameters

The choice of LoRA rank *r* plays a crucial role in balancing the model complexity and performance. As listed in Table 6, when *r* is set to 32 and 64, despite a slight reduction in *Params*, the model exhibits a significant drop of over 1% in *Dice* and *IoU*, indicating that excessively low r values hinder the model's feature extraction capabilities. In contrast, when a larger *r* is used, e.g., 256 and 512, no significant improvement of *Dice* and *IoU* is observed. Nevertheless, *Params* increases to 407M and 432M, with *GFLOPs* rising to 1,582 and 1,616, respectively. Notably, when *r* is set to 1,024, *Dice* and *IoU* increase by 0.1% to 0.3% compared to an *r* value of 128 at the price of 87M increment in *Params* and 122 in *GFLOPs*. Considering the training costs relative to the performance attained, the substantial increase in *Params* associated with an *r* value of 1,024 yields only marginal improvements in metrics. Therefore, the results suggest that setting *r* to 128 achieves favorable segmentation results without a significant increase in *Params*.

Table 7 presents the results of the ablation experiments on the configuration of EMA, focusing on scaling factors of token number and dimension. Due to the significant number of parameters introduced by Mamba, applying scaling on the token number and dimension helps to reduce the overall parameters of EMA. The ablation experiments with 9 different configurations of both scaling factors indicated that the optimal configuration is 3 for the token channel and 8 for the token dimension, and is used in the further analyses. As scaling factors increase, the *IoU* and *Dice* gradually decrease. These results indicated that large scaling factors can cause more loss of information, thereby hindering Mamba's ability to effectively extract features and negatively impacting performance. In addition, the token number is usually larger than the token dimension; a larger value of the scaling factor for the token number should be used. It is consistent with our experiment results.

### 5.4 Limitations and future work

Although the proposed SCMamba-SAM achieved significant improvements in medical image segmentation, especially for lesion segmentation, there are limitations that need to be stressed. First, single modality images are used in the proposed model, which would limit the performance in some segmentation tasks. Taking FCD lesion as an example, multi-modal images, e.g., T1, FLAIR, and PET, could provide complementary information to accurately characterize the lesions. Fusion of these multi-modality images into our model would be important to further improve segmentation performance and widen the application scope. Second, the model parameters and complexity increase significantly compared to SAM. Efforts should be made to design more lightweight architectures with comparable performances.

## 6 Conclusion

This article introduces a novel SCMamba-SAM framework for medical image segmentation. Based on the foundational SAM model, we designed a powerful foundational image encoder with LoRA fine-tuning technique, to customize the image encoder trained with natural images to medical images with fewer trainable parameters and lower complexity. A new adapter is proposed with the help of Mamba to capture global dependencies of tokens and channels, facilitating the adaptation of task-specific features. Extensive results on six public lesion segmentation datasets across different imaging modalities and body parts demonstrated that the proposed SCMamba-SAM achieved SOTA performance, exhibiting remarkable accuracy, and generalization. SCMamba-SAM holds promise as a useful tool for clinicians to diagnose and quantify lesions, providing important information for treatment decisions and surgery planning.

## Data Availability

The raw data supporting the conclusions of this article will be made available by the authors, without undue reservation.

## References

[1] Ker J Wang L Rao J Lim T. Deep learning applications in medical image analysis. IEEE Access. (2017) 6:9375–89. doi: 10.1109/ACCESS.2017.2788044

[2] Asgari Taghanaki S Abhishek K Cohen JP Cohen-Adad J Hamarneh G. Deep semantic segmentation of natural and medical images: a review. Artif Intell Rev. (2021) 54:137–78. doi: 10.1007/s10462-020-09854-1

[3] Isensee F Jaeger PF Kohl SAA Petersen J Maier-Hein KH. nnU-Net: a self-configuring method for deep learning-based biomedical image segmentation. Nat Methods. (2021) 18:203–11. doi: 10.1038/s41592-020-01008-z 33288961

[4] Khan S Naseer M Hayat M Zamir SW Khan FS Shah M. Transformers in vision: a survey. ACM Comput Surv. (2022) 54:1–41. doi: 10.1145/3505244

[5] Zhou HY Guo J Zhang Y Yu L Wang L Yu Y. nnformer: Interleaved transformer for volumetric segmentation. arxiv preprint arxiv:2109.03201 (2021).

[6] Hatamizadeh A Tang Y Nath V Yang D Myronenko A Landman B . Unetr: Transformers for 3d medical image segmentation. In: Proceedings of the IEEE/CVF winter conference on applications of computer vision (2022). p. 574–584. doi: 10.1109/WACV51458.2022.00181

[7] Cao H Wang Y Chen J Jiang D Zhang X Tian Q . Swin-unet: Unet-like pure transformer for medical image segmentation. In: European conference on computer vision. Cham: Springer Nature Switzerland (2022). p. 205–218. doi: 10.1007/978-3-031-25066-8_9

[8] Kirillov A Mintun E Ravi N Mao H Rolland C Gustafson L . Segment anything. In: Proceedings of the IEEE/CVF international conference on computer vision. (2023). p. 4015–4026. doi: 10.1109/ICCV51070.2023.00371

[9] Ji GP Fan DP Xu P Zhou B Cheng MM Van Gool L. SAM struggles in concealed scenes–empirical study on segment anything. arXiv preprint arXiv:2304.06022 (2023). doi: 10.1007/s11432-023-3881-x

[10] Zhou T Zhang Y Zhou Y Wu Y Gong C. Can SAM segment polyps? arXiv preprint arXiv:2304.07583 (2023).

[11] Huang Y Yang X Liu L Zhou H Chang A Zhou X . Segment anything model for medical images? Med Image Anal. (2024) 92:103061. doi: 10.1016/j.media.2023.103061 38086235

[12] Hu EJ Shen Y Wallis P Allen-Zhu Z Li Y Wang S . Lora:Low-rank adaptation of large language models. arXiv preprint arXiv:2106.09685 (2021).

[13] Ma J Li F Wang B. U-mamba: Enhancing long-range dependency for biomedical image segmentation. arxiv preprint arxiv:2401.04722 (2024).

[14] Liu J Yang H Zhou HY Xi Y Yu L Li C . Swin-umamba: Mamba-based unet with imagenet-based pretraining. In: International conference on medical image computing and computer-assisted intervention. Cham: Springer Nature Switzerland (2024). p. 615–625. doi: 10.1007/978-3-031-72114-4_59

[15] Dao T Gu A. Transformers are SSMS: generalized models and efficient algorithms through structured state space duality. arxiv preprint arxiv:2405.21060 (2024).

[16] Wu Y Zhang X Zhang H Sun Y Li L Zhu F . Mamba-SAM: an adaption framework for accurate medical image segmentation. In: IEEE international conference on bioinformatics and biomedicine (BIBM). IEEE (2024). p. 3856–9. doi: 10.1109/BIBM62325.2024.10821723

[17] Liang P Shi L Pu B Wu R Chen J Zhou L . MambaSAM: a visual mamba-adapted SAM framework for medical image segmentation. IEEE J Biomed Health Inform. (2025) 29:5824–5835. doi: 10.1109/JBHI.2025.3544548 40031635

[18] Ji W Li J Bi Q Liu T Li W Cheng L. Segment anything is not always perfect: an investigation of sam on different real-world applications. arXiv preprint arXiv:2304.05750 (2024).

[19] Ma J He Y Li F Han L You C Wang B. Segment anything in medical images. Nat Commun. (2024) 15:654. doi: 10.1038/s41467-024-44824-z 38253604 PMC10803759

[20] Zhang K Liu D. Customized segment anything model for medical image segmentation. arxiv preprint arxiv:2304.13785 (2023). doi: 10.2139/ssrn.4495221

[21] Wu J Wang Z Hong M Ji W Fu H Xu Y . Medical sam adapter: adapting segment anything model for medical image segmentation. arxiv preprint arxiv:2304.12620 (2023). 40121809 10.1016/j.media.2025.103547

[22] Cheng J Ye J Deng Z Chen J Li T Wang H . SAM-450 Med2D. arXiv preprint arXiv:2308.16184, (2023).

[23] Gu A Dao T. Mamba: Linear-time sequence modeling with selective state spaces. arxiv preprint arxiv:2312.00752 (2023).

[24] Gu A. Modeling Sequences with Structured State Spaces. Stanford University. (2023).

[25] Wang P Chung ACS. Focal dice loss and image dilation for brain tumor segmentation. In: Deep learning in medical image analysis and multimodal learning for clinical decision support: 4th international workshop, DLMIA 2018, and 8th international workshop, ML-CDS 2018, held in conjunction with MICCAI 2018, Granada, Spain, September 20, 2018, Proceedings 4. Springer International Publishing (2018). p. 119–127. doi: 10.1007/978-3-030-00889-5_14

[26] Codella NC Gutman D Celebi ME Helba B Marchetti MA Dusza SW . Skin lesion analysis toward melanoma detection: a challenge at the 2017 international symposium on biomedical imaging (ISBI), hosted by the international skin imaging collaboration (ISIC). In: 2018 IEEE 15th international symposium on biomedical imaging (ISBI 2018). IEEE (2018). p. 168–172. doi: 10.1109/ISBI.2018.8363547

[27] Codella N Rotemberg V Tschandl P Celebi ME Dusza S Gutman D . Skin lesion analysis toward melanoma detection 2018: a challenge hosted by the international skin imaging collaboration (isic). arXiv preprint arXiv:1902.03368 (2019).

[28] Jha D Smedsrud PH Riegler MA Halvorsen P De Lange T Johansen D . Kvasir-seg: a segmented polyp dataset. In: MultiMedia modeling: 26th international conference, MMM 2020, Daejeon, South Korea, January 5–8, 2020, proceedings, part II 26. Springer International Publishing (2020). p. 451–462. doi: 10.1007/978-3-030-37734-2_37

[29] Zhou J., et al. Thyroid nodule segmentation and classification in ultrasound images. In: *International conference on medical image computing and computer-assisted intervention*. (2020).

[30] Hssayeni M Croock M Salman A Al-Khafaji H Yahya Z Ghoraani B. Computed tomography images for intracranial hemorrhage detection and segmentation. PhysioNet. (2020) 5:1–4. doi: 10.3390/data5010014

[31] Schuch F Walger L Schmitz M David B Bauer T Harms A . An open presurgery MRI dataset of people with epilepsy and focal cortical dysplasia type II. Sci Data. (2023) 10:475. doi: 10.1038/s41597-023-02386-7 37474522 PMC10359264

[32] Kingma DP Ba J. Adam: a method for stochastic optimization. arxiv preprint arxiv:1412.6980 (2014).

[33] Dice LR. Measures of the amount of ecologic association between species. Ecology. (1945) 26:297–302. doi: 10.2307/1932409

[34] Everingham M Van Gool L Williams CK Winn J Zisserman A. The pascal visual object classes (voc) challenge. Int J Comput Vis. (2010) 88:303–38. doi: 10.1007/s11263-009-0275-4

[35] Powers DMW. Evaluation: from precision, recall and F-measure to ROC, informedness, markedness and correlation. arxiv preprint arxiv:2010.16061 (2020).

[36] Ronneberger O Fischer P Brox T. U-net: convolutional networks for biomedical image segmentation. In: Medical image computing and computer-ssisted intervention–MICCAI 2015: 18th international conference, Munich, Germany, October 5–9, 2015, proceedings, part III 18. Springer International Publishing (2015). p. 234–241. doi: 10.1007/978-3-319-24574-4_28

[37] Peng Y Sonka M Chen DZ. U-Net v2: rethinking the skip connections of U-Net for medical image segmentation. arxiv preprint arxiv:2311.17791 (2023).

[38] Alom MZ Hasan M Yakopcic C Taha TM Asari VK. Recurrent residual convolutional neural network based on u-net (r2u-net) for medical image segmentation. arxiv preprint arxiv:1802.06955 (2018).

[39] Wu H Chen S Chen G Wang W Lei B Wen Z. FAT-Net: feature adaptive transformers for automated skin lesion segmentation. Med Image Anal. (2022) 76:102327. doi: 10.1016/j.media.2021.102327 34923250

[40] Beisser D Klau GW Dandekar T Muller T Dittrich MT. BioNet: an R-Package for the functional analysis of biological networks. Bioinformatics. (2010) 26:1129–30. doi: 10.1093/bioinformatics/btq089 20189939

[41] Chen LC Papandreou G Schroff F Adam H. Rethinking atrous convolution for semantic image segmentation. arXiv preprint arXiv:1706.05587 (2017).

[42] Xie Y Zhang J Xia Y Shen C. A mutual bootstrapping model for automated skin lesion segmentation and classification. IEEE Trans Med Imaging. (2020) 39:2482–93. doi: 10.1109/TMI.2020.2972964 32070946

[43] Xie Y Zhang J Lu H Shen C Xia Y. SESV accurate medical image segmentation by predicting and correcting errors. IEEE Trans Med Imaging. (2020) 40:286–96. doi: 10.1109/TMI.2020.3025308 32956049

[44] Chen J Lu Y Yu Q Luo X Adeli E Wang Y . Transunet: transformers make strong encoders for medical image segmentation. arxiv preprint arxiv:2102.04306 (2021).

[45] Yang G Yu S Dong H Slabaugh G Dragotti PL Ye X . DAGAN: deep de-aliasing generative adversarial networks for fast compressed sensing MRI reconstruction. IEEE Trans Med Imaging. (2017) 37:1310–21. doi: 10.1109/TMI.2017.2785879 29870361

